# Therapeutic potential of oral alginate nanoparticles against experimental toxoplasmosis

**DOI:** 10.1007/s00436-024-08307-w

**Published:** 2024-08-06

**Authors:** Hoda A. Rashed, Amany Abdel-Bary, Eman A. Elmorsy

**Affiliations:** 1 Medical Parasitology Department, Faculty of Medicine, Alexandria University, Alexandria, Egypt; 2 Pathology Department, Faculty of Medicine, Alexandria University, Alexandria, Egypt

**Keywords:** Alginate nanoparticles, RH *Toxoplasma* strain, IFN-γ, EM, Histopathology

## Abstract

Side effects and low efficacy of current anti-toxoplasmosis therapeutics against encysted bradyzoites necessitate research into alternative safe therapeutic options. The safety, immunostimulatory, and antimicrobial properties of alginate nanoparticle formulation (Alg-NP) highlight its potential as an oral therapy against acute toxoplasmosis. In the current study, Alg-NP was formulated and characterized and then assessed for its anti-Toxoplasma effects using parasitological, ultrastructural, immunological, and histopathological studies. Treatment with Alg-NP significantly prolonged mice survival and reduced the parasite burden in both peritoneal fluid and tissue impression smears. In addition, it altered parasite viability and caused severe tachyzoite deformities as evidenced by ultrastructural studies. Alg-NP induced high levels of serum IFN-γ in infected mice with significant amelioration in histopathological changes in both hepatic and splenic tissue sections. In conclusion, Alg-NP could be considered a promising therapeutic agent against acute murine toxoplasmosis, and owing to its safety, it could potentially be enlisted for human use.

## Background

Toxoplasma is a cosmopolitan intracellular apicomplexan parasite that infects nearly one-third of the human population (Desmettre 2020). Humans can acquire infection via ingestion of mature sporulated oocysts shed by felines in contaminated water or food. Infection can also be acquired by ingestion of dormant bradyzoite cysts in undercooked meat. In immunocompetent subjects, toxoplasmosis is usually asymptomatic, or may cause mild self-limiting flu-like manifestations, with or without lymphadenopathy. In immunocompromised patients however, Toxoplasma is considered a significant threat causing severe ocular and neurological manifestations. Congenital toxoplasmosis is not uncommon and can lead to severe neurological deformities (Cañón-Franco et al. 2014; El-Zawawy et al. 2015).

Currently, the mainstay treatment of toxoplasmosis depends on the synergistic combination of pyrimethamine and sulfadiazine, trimethoprim-sulfamethoxazole (TMP-SMZ), or triple sulfonamides. However, these regimens cause severe side effects such as bone marrow suppression, hematological disorders, and severe allergic reactions. Alternative therapies such as clarithromycin, azithromycin, artemisinin, dapsone, and atovaquone are less effective against bradyzoites (Cañón-Franco et al. 2014; El-Zawawy et al. 2015). Several experimental trials have been conducted to reach an effective therapeutic regimen for toxoplasmosis, but few have reached clinical trials.

Using nanotechnology is a great advance in the treatment of different parasitic diseases. Besides the antimicrobial effects, nanoparticles improve drug availability at the target site and minimize drug toxicity (AlGabbani 2023; Khezerlou et al. 2018). Many trials have been conducted using different types of nanoparticles to treat toxoplasmosis (El-Zawawy et al. 2015; Gaafar et al. 2014). One of these trials used alginate nanoparticle (Alg-NP) as a delivery system for spiramycin, resulting in a significant reduction of the parasite burden and an improvement in mice survival (Hagras et al. 2022).

Alginate is a natural biopolymer that constitutes a structural component in marine brown algae and capsular polysaccharides in some bacteria (Kennedy and Hewlett 2007). It is extracted by the basic neutralization of alginic acid from brown algae (Lee and Mooney 2012). Alginate hydrogels have been used for several biomedical applications, such as wound healing and as a drug delivery system for antibacterial and antineoplastic agents (Lee and Mooney 2012; Rao et al. 2014). Alginate nanoparticles are polymeric nanoparticles with great safety, suitable bioavailability, and easy preparation (Draget and Taylor 2011; Torres-Sangiao et al. 2016). Furthermore, the immunogenic properties of alginate have been documented by its ability to induce pro-inflammatory cytokines such as tumor necrosis factor-α (TNF-α) and activation of CD4 lymphocytes (Draget and Taylor 2011). Hence, the objective of the current study was to assess the therapeutic effect of Alg-NP against acute experimental toxoplasmosis.

## Material and methods

### Parasite

*Toxoplasma gondii* (*T. gondii*) virulent RH strain was obtained and maintained in the laboratory of the Medical Parasitology Department, Alexandria Faculty of Medicine, Egypt, by serial intraperitoneal (IP) passage of tachyzoites. Five days after infection, tachyzoites were collected by peritoneal lavage and kept in phosphate-buffered saline (PBS). Tachyzoites were then used for animal infection at a dose of 2500 parasites per mouse (Gaafar et al. 2022).

### The tested agents

#### Trimethoprim-sulfamethoxazole (Septrin ®)

Oral suspension of TMP-SMZ (Septrin®, GlaxoSmithKline) was purchased from a local pharmacy and used as a therapeutic control (Hagras et al. 2022).

#### Alginate nanoparticle preparation and characterization

The ionic gelation method was used to formulate Alg-Np. A sodium alginate aqueous solution was prepared by dissolving 20 mg of sodium alginate powder in 10 ml of bi-distilled water. Ten milligrams of calcium chloride dihydrate was dissolved in 10 ml of distilled water to form a calcium chloride aqueous solution. Then, calcium chloride aqueous solution was added to sodium alginate solution dropwise under a constant stirring rate of 1300 rpm for 45 min at room temperature. Later, the NP pellet was separated via centrifugation at 30,000 × g for 30 min at 4 °C and the supernatant was discarded. The precipitated NP was lyophilized and weighed, then stored at 4 °C until use (Gaafar et al. 2022; Sarei et al. 2013).

The morphology and size of the prepared NP were examined by scanning electron microscopy (SEM) (JEOL. JSM, 6360LA, Japan) and transmission electron microscopy (TEM) (JEOL-100 CX, Japan). The hydrodynamic size, polydispersity index (PDI), and zeta potential (ζ) were measured by a nano-zeta sizer (Nano-ZS, Malvern Co., UK), which determines the diameter of the particles using the dynamic light scattering technique (DLS). The measurements were carried out in disposable polystyrene cuvettes at 25 °C with measurement angles of 12.5° and 170° (Gaafar et al. 2022).

### Experimental animals and study design

Laboratory-bred male Swiss albino mice aged 3 to 5 weeks and weighing 20 to 25 g were the subject of the study. They were housed in separate cages in a well-ventilated room in the animal house of the Medical Parasitology Department, Alexandria Faculty of Medicine. They were bred on a commercial diet of fibers and proteins, and their bedding was changed daily. Mice stools were examined and reported to be free from any parasitic infections.

Forty-eight mice were allocated into two main groups. Group I (control group) included 24 mice that were further subdivided into three subgroups as follows: subgroup Ia, 6 non-infected non-treated mice; subgroup Ib, 6 non-infected, and Alg-NP-treated mice; and subgroup Ic, 12 infected non-treated control mice. Group II (experimental group) included 24 infected and treated mice that were further subdivided into two equal subgroups: subgroup IIa (TMP-SMZ-treated) and subgroup IIb (Alg-NP-treated).

### Animal infection and treatment schedule

Mice infection was carried out by IP injection of each mouse by 2500 tachyzoites of a virulent RH strain (Hagras et al. 2022). The TMP-SMZ combination was given at a dose of 100 mg/kg/day, and the Alg-NP formulation was given at a dose of 20 mg/kg/day (FarahatAllam et al. 2020; Rageh et al. 2022). Treatment regimens started 4 h after the infection, orally using a gavage needle. Dosages were repeated once daily for seven consecutive days (Gomaa and Sheta 2022). Six mice from each infected subgroup (Ic, IIa, and IIb) were sacrificed on the 7th day post-infection to assess the tested regimens. The remaining six mice in each subgroup were observed daily to estimate the survival time.

### Evaluation of drug efficacy

The efficacy of Alg-NP was evaluated and compared to TMP-SMZ by parasitological, ultrastructural, immunological, and histopathological studies.

### Parasitological study

#### Survival time

Daily recording of the mortality of infected mice was done until the death of all mice.

#### Tachyzoites count

Tachyzoites were counted in smears from the peritoneal lavage fluid using a hemocytometer and the × 400 objective lens of an ordinary light microscope. Extracellular parasites in hepatic and splenic impression smears were counted in ten different oil immersion fields on each slide, and the mean count was then calculated (Gaafar et al. 2022).

#### Parasite viability

Tachyzoites collected from the peritoneal fluid of infected subgroups were stained with 0.4% trypan blue stain (w/v) (Chorawala et al. 2013). The dead parasites were detected by dark blue staining and unrecognized internal structures, while the viable ones showed dye exclusion activity and appeared clear with light blue staining. The percentage of live tachyzoites in each mouse was estimated microscopically using a × 400 objective, and the mean number of live tachyzoites in each subgroup was calculated (El-Zawawy et al. 2015; Gaafar et al. 2022).

Parasite burden and viability reduction were calculated according to the following formula:

$$Percentage reduction (\%R)=100-[(n\div N)\times 100]$$

where (*n*) is the mean parasite count or viability in each infected treated subgroup and (*N*) is the mean parasite count or viability in the infected non-treated control subgroup.

### Ultrastructural study

Peritoneal exudate collected from infected non-treated and Alg-NP-treated subgroups after sacrifice was fixed in 2.5% glutaraldehyde and prepared for further parasite ultrastructure examination using SEM and TEM (Gaafar et al. 2022).

### Immunological and biochemical study

Blood samples collected before sacrifice were left to clot at room temperature, then centrifuged at 2000 × g for 20 min. Serum samples were aliquoted and stored at − 20 °C until use. The level of interferon-gamma (INF-γ) was measured in the sera of all studied subgroups by ELISA kits (Biospes Co., Ltd.) according to the manufacturer’s instructions. Part of the sera from subgroups Ia and Ib was used for the biochemical study to demonstrate the safety of Alg-NP on liver and kidney functions. Aspartate transaminase (AST), alanine transaminase (ALT), and alkaline phosphatase (ALP) were measured as markers for hepatic cell integrity, and serum urea and creatinine were assayed for kidney function (Chorawala et al. 2013).

### Histopathological study

Specimens from the liver and the spleen of the infected treated group and the infected non-treated control subgroup were fixed in 10% neutral formalin, sectioned, and stained with hematoxylin–eosin (H and E) stain to assess histologic alteration in different groups (Musumeci 2014).

### Statistical analysis

Results were analyzed using IBM SPSS software package version 20.0. (Armonk, NY: IBM Corp.). For continuous data, normality was tested by the Shapiro–Wilk test. Quantitative data were expressed as range (minimum and maximum), mean, and standard deviation. A Student *t*-test was used to compare two subgroups for normally distributed quantitative variables, while a one-way ANOVA test was used for comparing more than two studied subgroups, followed by the post hoc test (Tukey) for pairwise comparison. The significance of the obtained results was judged at the 5% level. A Kaplan–Meier survival curve and log-rank test were done for the significant relationship with animal survival.

## Results

### Alginate nanoparticle characterization (Fig. 1)

**Fig. 1:**
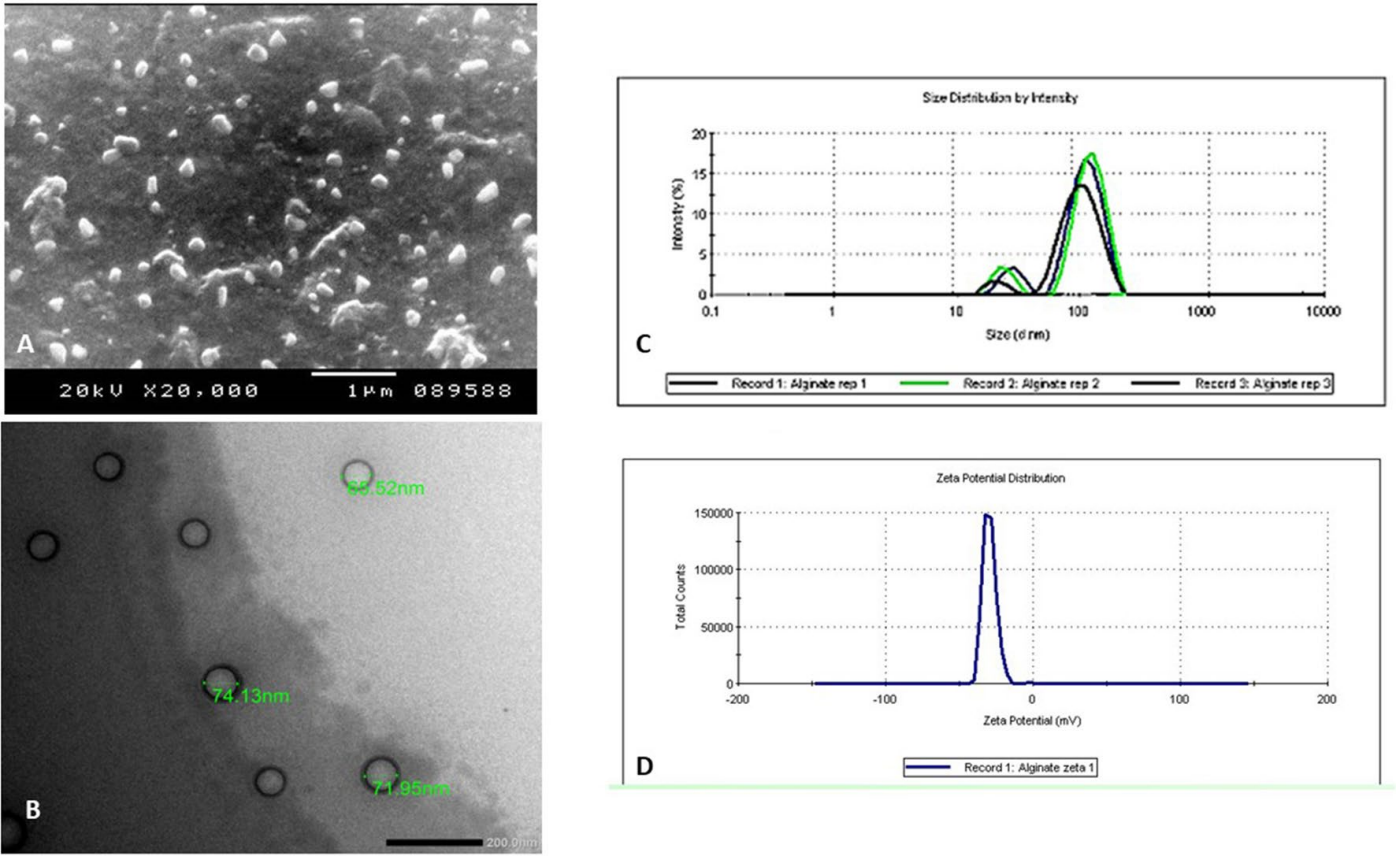
Characterization of Alg-NP. A SEM of Alg-NP (× 20,000). B TEM of blank Alg-NP (× 25,000). C Particle size distribution of Alg-NP. D Zeta potential of Alg-NP

Ultrastructural characterization showed the prepared Alg-NP as spherical or oval particles with a smooth regular outline. Their mean diameter was 70.53 ± 4.31 nm. The nanoparticles were dispersed with no observed aggregation (Figs. 1A and B). Using a zeta-sizer, the Alg-NP formulation was homogenous with a PDI of 0.402 and a mean hydrodynamic particle size of 188.44 ± 20.19 nm (Fig. 1C). The formulated Alg-NP had a zeta potential of − 32.1 mV as determined by dynamic light scattering (Fig. 1D).

### Parasitological results

#### Survival time (Fig. 2)

**Fig. 2:**
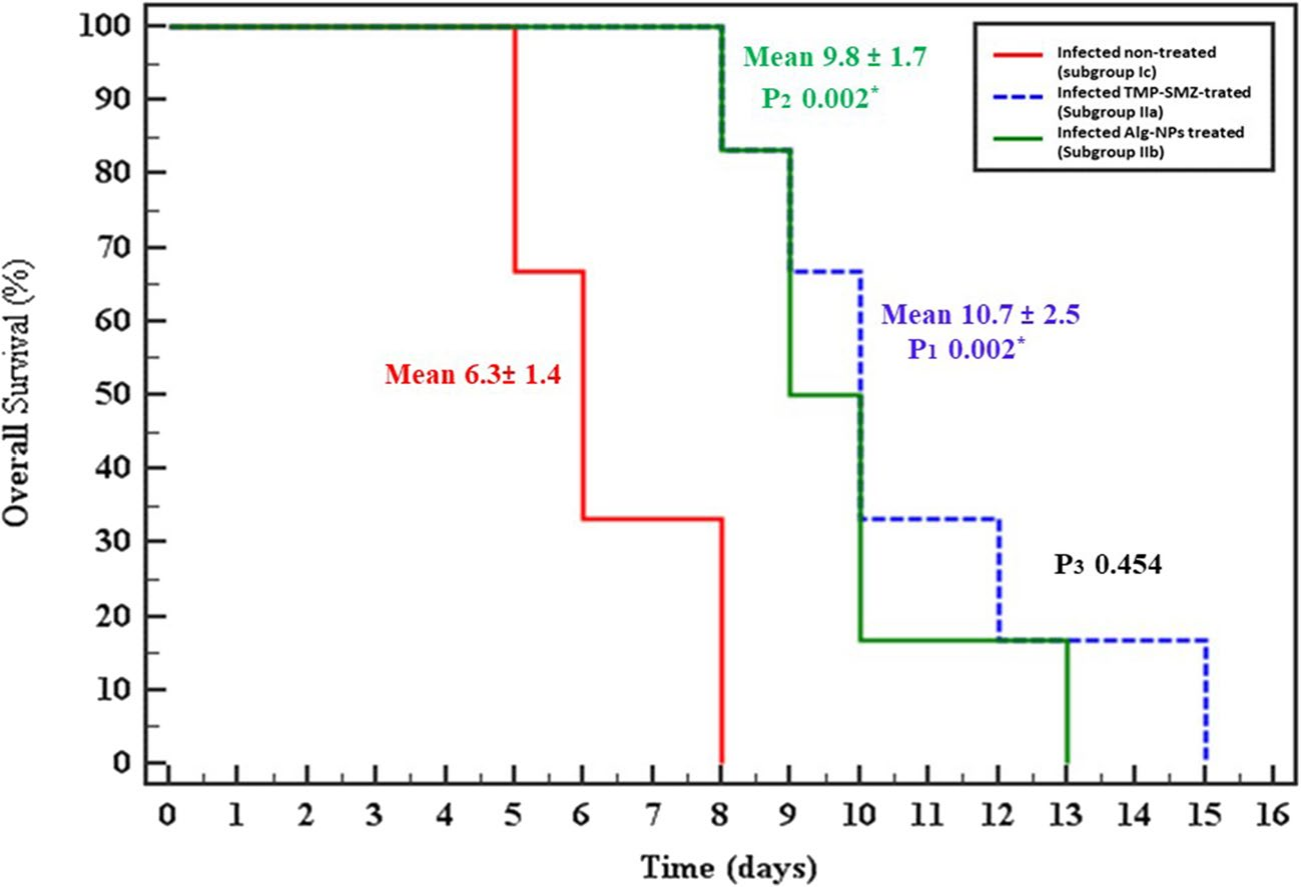
Kaplan–Meier survival curve in different studied groups. Subgroup Ic: infected non-treated control; subgroup IIa: infected TMP-SMZ-treated; subgroup IIb: infected Alg-NP-treated. A log-rank test was done for the significant relation with animal survival: p_1_: *p*-value for comparing between subgroup Ic and subgroup IIa; p_2_: *p*-value for comparing between subgroup Ic and subgroup IIb; p_3_: *p*-value for comparing between subgroup IIa and subgroup IIb; *: statistically significant at *p* ≤ 0.05

The mean survival time in the infected, non-treated control subgroup (Ic) was 6.3 ± 1.4 days. Statistically, a significant difference was noted between the latter subgroup and the infected-treated subgroups (IIa and IIb). Interestingly, Alg-NP treatment extended mice survival time up to 13 days post-infection compared to maximal survival of 8 days in the infected non-treated subgroup. However, a non-significant difference was noted between the TMP-SMZ-treated subgroup (IIa) and the Alg-NP-treated subgroup (IIb) (Fig. 2).

#### Parasite count (Fig. 3; Table 1)

**Fig. 3:**
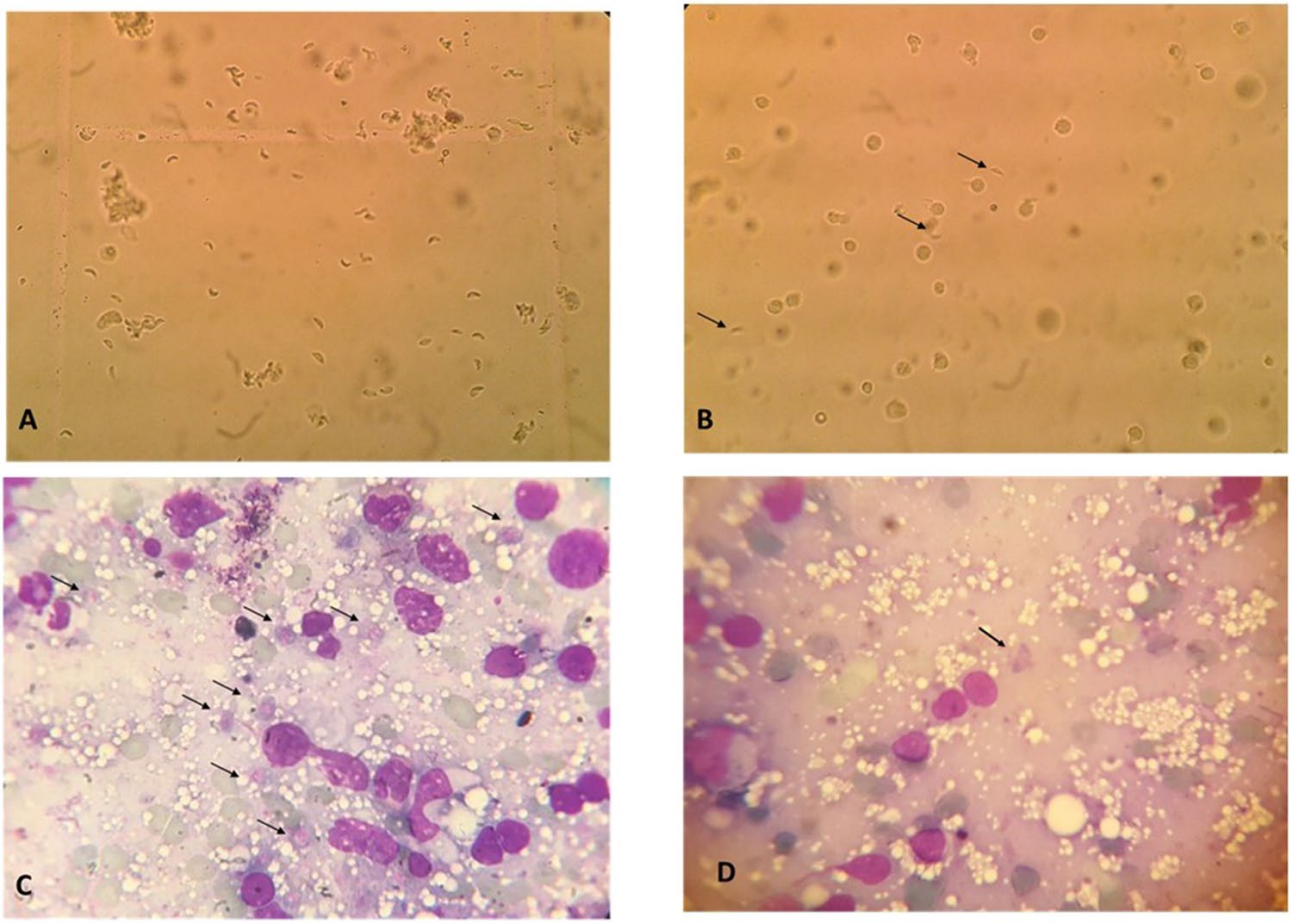
*Toxoplasma* tachyzoites count in peritoneal fluid lavage and Giemsa-stained impression smears. A Many tachyzoites in peritoneal fluid lavage of the infected non-treated control subgroup using a hemocytometer (× 400). B Few tachyzoites in peritoneal fluid lavage of the Alg-NP-treated control subgroup (arrows) using a hemocytometer (× 400). C Tachyzoites in an impression smear of an infected non-treated control subgroup (arrows) (Giemsa stain × 1000). D Tachyzoites in an impression smear of an infected Alg-NP-treated subgroup (arrows) (Giemsa stain × 1000)

**Table 1:** Tachyzoite count in the different infected subgroups

	Infected nontreated (subgroup Ic)	Infected TMP-SMZ treated (subgroup IIa)	Infected Alg-NP treated (subgroup IIb)	F
Peritoneal fluid count (10^4^)				114.334^*^
Min.–max	104–176	4–13	10–32	
Mean ± SD	135.3 ± 25.9	7.8^a^ ± 2.9	22^a^ ± 9.4	
Significance	*p*_1_ < 0.001^*^, *p*_2_ < 0.001^*^, *p*_3_ = 0.304			
% Reduction		94.24	83.73	
Liver impression count				74.935*
Min.–max	12.6–23	0–4.6	2–5.6	
Mean ± SD	18.2 ± 3.9	1.7^a^ ± 1.7	3.8^a^ ± 1.3	
Significance	*p*_1_ < 0.001^*^, *p*_2_ < 0.001^*^, *p*_3_ = 0.368			
% Reduction		90.66	79.12	
Spleen impression count				101.514
Min.–max	10–17.6	0–3	0–2.3	
Mean ± SD	13.6 ± 2.6	1.3^a^ ± 1.1	1.5^a^ ± 0.80	
Significance	*p*_1_ < 0.001^*^, *p*_2_ < 0.001^*^, *p*_3_ = 0.978			
% Reduction		90.44	88.97	

Pairwise comparison bet. each 2 subgroups was done using post hoc test (Tukey)

*F*, F for one-way ANOVA test; *p1*, *p* value for comparing between subgroup Ic and subgroup IIa; *p2*, *p* value for comparing between subgroup Ic and subgroup IIb; *p3*, *p* value for comparing between subgroup IIa and subgroup IIb

^*^Statistically significant at *p* ≤ 0.05

^a^Significant with subgroup Ic

^b^Significant with subgroup IIa

The parasite counts in peritoneal lavage fluid showed a statistically significant reduction in treated subgroups (IIa, IIb) compared to the non-treated control group. Despite the 83.73% reduction in the Alg-NP-treated subgroup (IIb), this was non-statistically significant versus the TMP-SMZ-treated subgroup. Similarly, a significant reduction of 79.12% in liver impression smears and 88.97% in spleen impression smears from subgroup IIb versus control group Ic was detected, and a non-significant difference compared to treated subgroup IIa.

#### Parasite viability (Table 2)

**Table 2:** Viable tachyzoite% among different infected subgroups

	Infected nontreated (subgroup Ic)	Infected TMP-SMZ treated (subgroup IIa)		Infected Alg-NP treated (subgroup IIb)	F
Viable tachyzoite %					
Min.–max	95–100	27–43	40–60		214.680*
Mean ± SD	98.7 ± 2	34.7^a^ ± 5.9	52.3^ab^ ± 7.3		
% reduction		64.8	47.1		
Significance	*p*1 < 0.001*, *p*2 < 0.001*, *p*3 < 0.001*				

Pairwise comparison between each 2 subgroups was done using post hoc test (Tukey)

*F*, F for one-way ANOVA test; *p1*, *p* value for comparing between subgroup Ic and subgroup IIa; *p2*, *p* value for comparing between subgroup Ic and subgroup IIb; *p3*, *p* value for comparing between subgroup IIa and subgroup IIb

^*^Statistically significant at *p* ≤ 0.05

^a^Significant with subgroup Ic

^b^Significant with subgroup IIa

Both the TMP-SMZ-treated subgroup (IIa) and the Alg-NP-treated subgroup (IIb) showed a statistically significant reduction in the percentage of viable parasites compared to the infected control subgroup (Ic), with a reduction of 64.8% and 47.1%, respectively. However, there is a statistically significant difference between TMP-SMZ-treated and Alg-NP-treated subgroups on the side of the former.

### Ultrastructural results (Fig. 4)

**Fig. 4:**
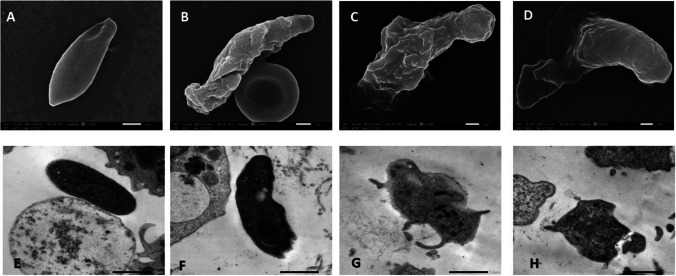
Electron microscopic study of tachyzoites. A SEM of tachyzoite collected from an infected non-treated control subgroup showing well-preserved crescent-shaped tachyzoite with a smooth, intact membrane and apparent conoid (× 15,000). B–D SEM of tachyzoite recovered from the Alg-NP-treated subgroup. B Tachyzoite showing profound morphological alterations in the form of surface irregularities with multiple furrows and deep clefts (× 12,000). C and D Tachyzoite showing extensive deformities and distortion of its membrane with leakage of cytoplasmic content (× 11,000 and 10,000). E TEM of tachyzoite from the infected control subgroup showing a normal crescent shape and intact cellular and nuclear membranes with prominently dense granules in an electron-dense matrix (× 8000). F–H TEM of tachyzoite in the Alg-NP-treated subgroup. F Tachyzoite showing extensive cytoplasmic vacuolation, clefting of the nuclear membrane with condensed chromatin, and irregularities associated with cell membrane disruption (× 8000). G and H Tachyzoites had lost their structure completely, with profound damage to all organelles (× 8000)

SEM analysis showed that tachyzoites collected from the infected control subgroup displayed a well-preserved crescent shape with a smooth, intact membrane and evident conoid (Fig. 4A). On the other side, tachyzoites retrieved from the Alg-NP-treated subgroup exhibited profound morphological alterations in the form of surface irregularities with multiple furrows and deep clefts (Fig. 4B). Besides, distortion of the tachyzoite membrane with leakage of cytoplasmic content was noted, as shown in Figs. 4C and D.

TEM demonstrated the tachyzoites retrieved from the infected control subgroup with intact cellular and nuclear membranes and prominent dense granules in an electron-dense matrix (Fig. 4E), whereas tachyzoites collected from the Alg-NP-treated subgroup showed extensive cytoplasmic vacuolation, evident clefting of the nuclear membrane with condensed chromatin, and irregularities associated with cell membrane disruption (Fig. 4F). Moreover, tachyzoites lost their integral structure with profound damage to all organelles (Fig. 4G and H).

### Immunological and biochemical results (Fig. 5; Table 3)

**Fig. 5:**
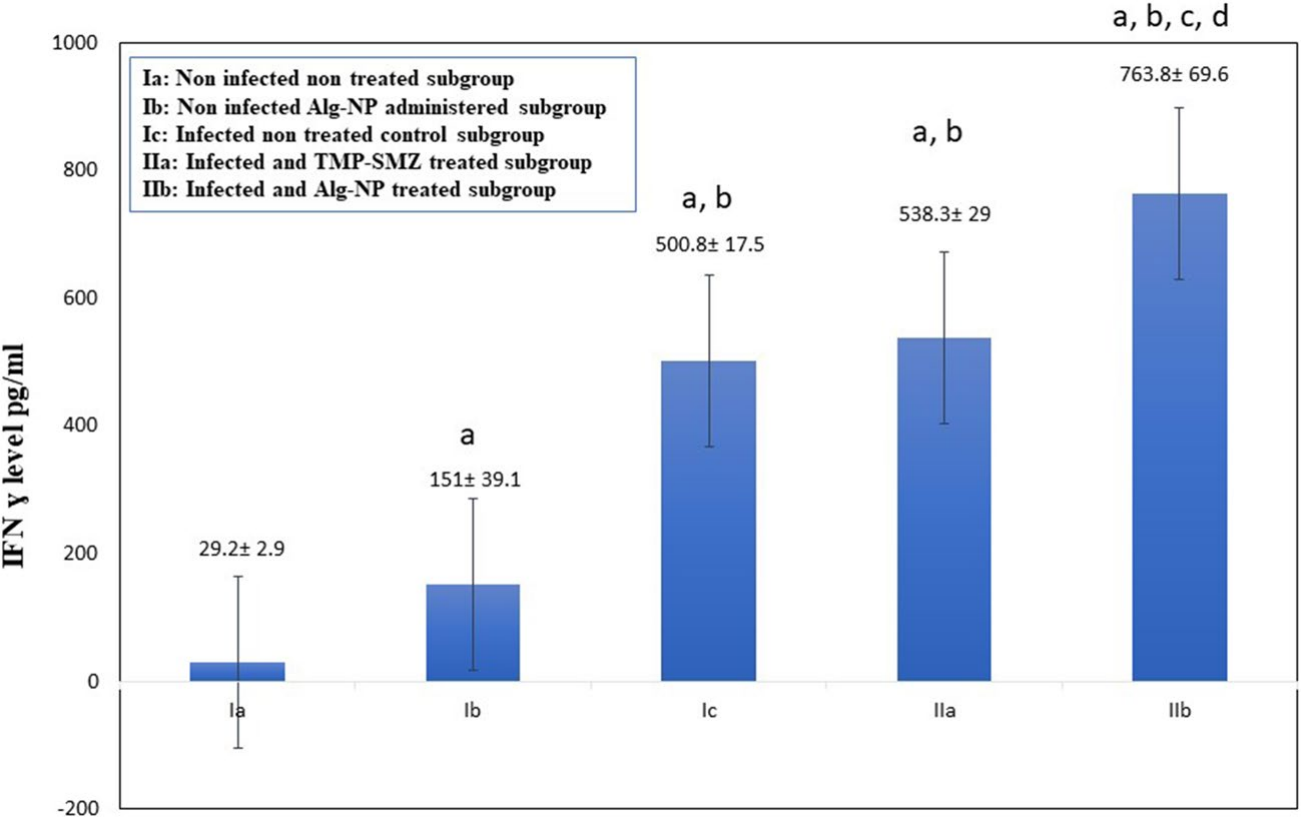
The INF-γ level among the different studied subgroups. Ia, non-infected non-treated subgroup; Ib, non-infected Alg-NP administered subgroup; Ic, infected non-treated control subgroup; IIa, infected and TMP-SMZ-treated subgroup; IIb, infected and Alg-NP-treated subgroup. F, F for the one-way ANOVA test. Pairwise comparison between each group was done using the post hoc test (Tukey). a, significant with Ia; b, significant with Ib; c, significant with Ic; d, significant with IIa. Statistically significant at *p* ≤ 0.05

**Table 3:** Liver enzymes and kidney function tests among non-infected subgroups

	Non-infected non-treated (subgroup Ia)	Non-infected Alg-NP treated (subgroup Ib)
ALT(IU/L)		
Min.–max	53.9–61.3	56.3–62.5
Mean ± SD	57.2 ± 2.9	59.4 ± 2.6
AST(IU/L)		
Min.–max	248.9–302.2	268.2–306.6
Mean ± SD	274.6 ± 23.1	286.9 ± 4
ALP (IU/L)		
Min.–max	158–183	162–195.4
Mean ± SD	171.2 ± 9.5	180.5 ± 8.1
BUN (mmol/L)		
Min.–max	9.6–11.2	10.1–11.4
Mean ± SD	10.2 ± 0.59	10.7 ± 0.60
CRE (mmol/L)		
Min.–max	30.5–38.1	32.7–37.57
Mean ± SD	34.78 ± 2.9	35.17 ± 2.1

There is no statistically significant difference between two subgroups

*ALT*, alanine transaminase; *AST*, aspartate transaminase; *ALP*, alkaline phosphatase; *BUN*, serum urea; *CRE*, serum creatinine

Immunologically, a statistically significant increment in serum INF-γ level was noted in Alg-NP-treated mice in subgroup Ib compared to the non-infected control subgroup (Ia), as depicted in Fig. 5. Both infected and treated subgroups (IIa, IIb) had statistically significant higher levels of serum INF-γ in comparison to the infected, non-treated control subgroup (Ic). Besides, the Alg-NP-treated subgroup (IIb) demonstrated a comparable statistically significant increase in serum INF-γ level to the TMP-SMZ-treated subgroup (IIa).

Biochemical assays highlighted the safety of the used Alg-NP formulation, where non-significant differences were detected in the markers of liver and kidney functions in normal mice (Ia) and Alg-NP-treated mice in subgroup Ib.

### Histopathological results (Figs. 6 and 7)

**Fig. 6:**
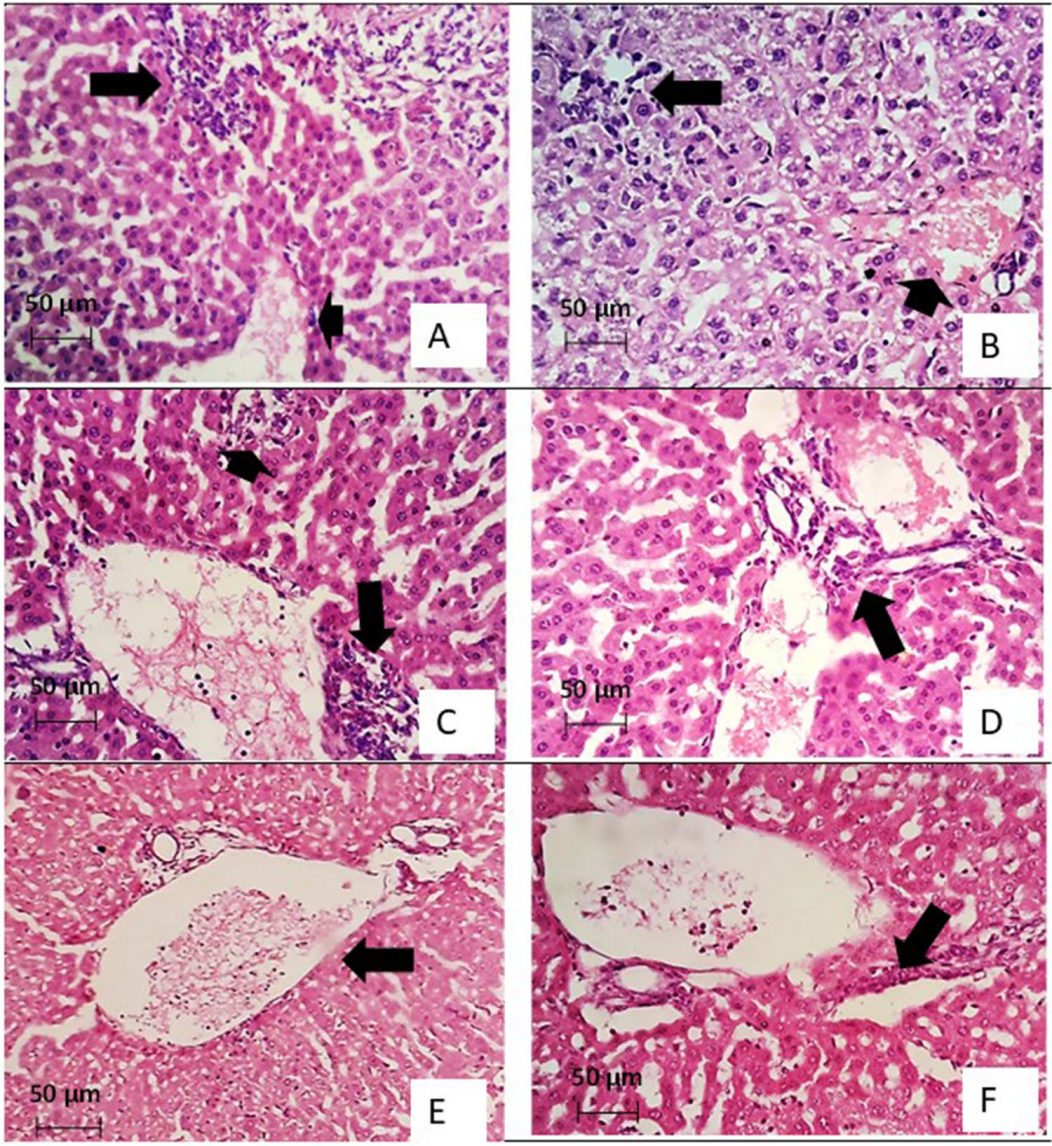
H&E-stained sections of hepatic tissues in *Toxoplasma-*infected subgroups. A Liver section of the infected control subgroup showing mild distortion of the hepatic architecture, congested central vein (arrowhead), and moderate lymphocytic infiltration (arrow). B Liver section of the infected control subgroup showing mild distortion of the liver architecture, congested, and expanded central vein (arrowhead) with a tissue cyst containing tachyzoites (arrow). C Liver section of the infected and TMP-SMZ-treated subgroup showing preserved liver architecture with peri-central lymphocytic infiltration (arrow) and a cyst showing tachyzoites (arrowhead). D Liver section of the infected and TMP-SMZ-treated subgroup showing preserved liver architecture with a dilated, expanded central vein and portal tract (arrow) but no tachyzoites. E Liver section of the infected Alg-NP-treated subgroup showing no tachyzoites with a less congested central vein (arrow). F Liver section of an infected Alg-NP-treated subgroup showing mild periportal lymphocytic infiltration (arrow) with no tachyzoites

**Fig. 7:**
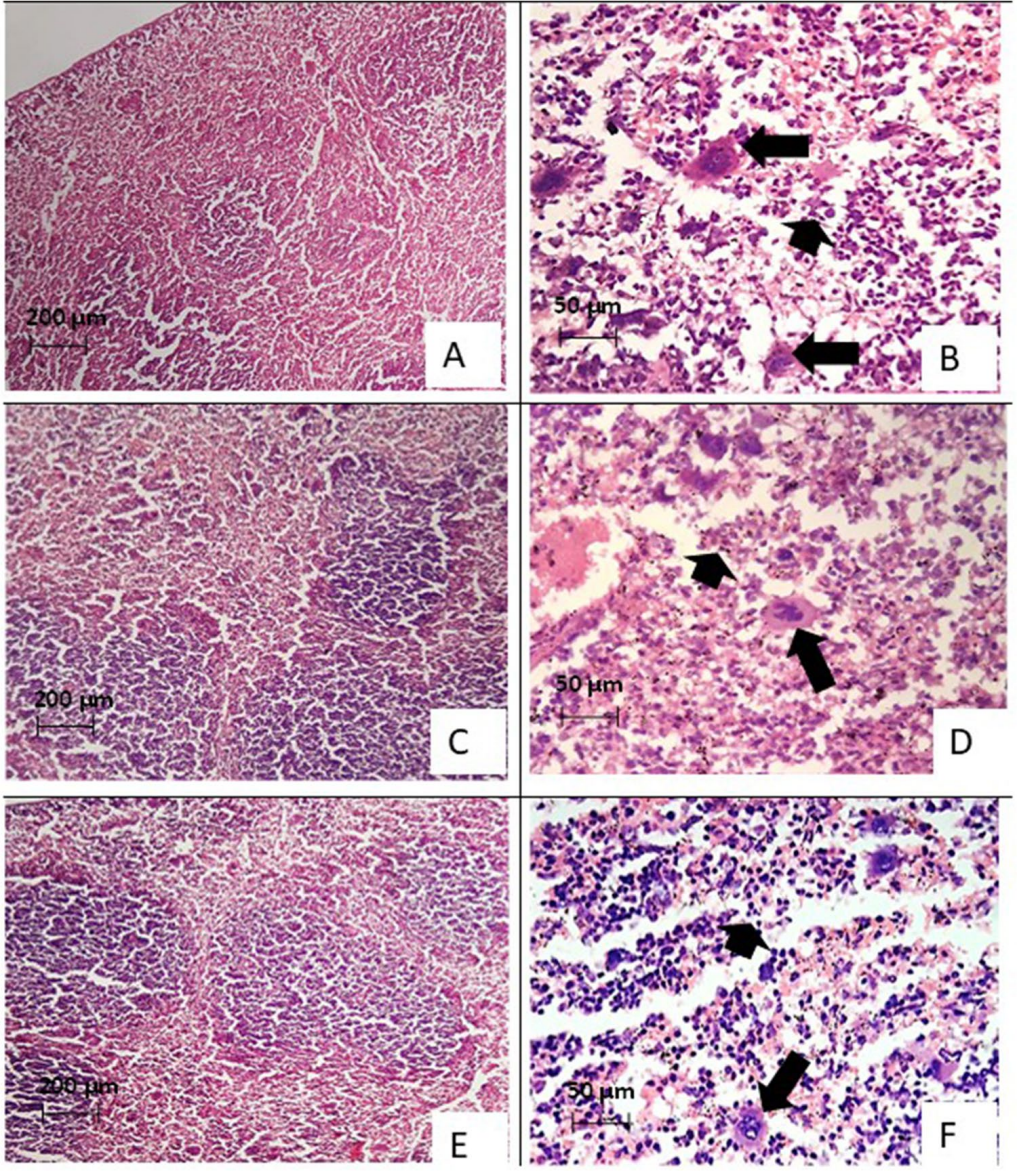
H&E-stained sections of splenic tissues in *Toxoplasma-*infected subgroups. A Splenic section of infected control subgroup showing congestion and atrophic white pulp with expanded red pulp. B Splenic section of infected control subgroup with numerous reactive multinucleated giant cells engulfing numerous tachyzoites and surrounded by inflammatory cells with excess apoptosis (arrowhead). C Splenic section of infected and TMP-SMZ-treated subgroup showing mild atrophy of the white pulp. D Splenic section of infected and TMP-SMZ-treated subgroup showing some multinucleated large cells loaded with some tachyzoites (arrow) associated with moderated apoptosis (arrowhead). E Splenic section of infected Alg-NP-treated subgroup showing preserved white pulp with hyperplastic lymphoid follicles. F Splenic section of infected Alg-NP-treated subgroup showing scattered multinucleated giant cells loaded with few tachyzoites (arrow) in association with mild apoptosis (arrowhead)

Histopathological examination of liver sections of the infected non-treated control subgroup (Ic) showed mild distortion of liver architecture with heavy infiltration of the parenchyma with inflammatory cells, together with hyperplasia of parasite-loaded kupffer cells. Severe congestion of the portal tract and the central vein was noted with multiple foci of necrosis associated with fibrosis and expansions (Fig. 6A and B).

On the other hand, improvement of hepatic pathological changes was evident in treated subgroups IIa and IIb. A preserved liver architecture was noted, with few foci of necro-inflammatory infiltrate that were less densely loaded with tachyzoites in TMP-SMZ treated subgroup (Fig. 6C and D). In Alg-NP treated subgroup, minimal parasites were detected with mild periportal lymphocytic infiltrate and less congestion. Besides, neither necrotic nor fibrotic foci were noted (Figs. 6E and F).

Similarly, splenic sections showed amelioration of histopathological changes in subgroups IIa and IIb compared to architectural derangements observed in subgroup Ic. The infected control subgroup showed splenic congestion and atrophic white pulp with expanded red pulp. Splenic tissue was infiltrated with numerous reactive multinucleated giant cells that were heavily loaded with tachyzoites and surrounded by inflammatory cells with excess apoptosis (Figs. 7A and B).

Splenic sections of the TMP-SMZ-treated subgroup demonstrated mild atrophy of the white pulp and less multinucleated giant cells loaded with tachyzoites. This was associated with moderate apoptosis (Figs. 7C and D). On the other hand, splenic sections of the Alg-NP treated subgroup showed preserved white pulp with hyperplastic lymphoid follicles (Fig. 7E). Scattered multinucleated giant cells loaded with scanty tachyzoites were observed and were surrounded by mild inflammatory cells associated with mild apoptosis (Fig. 7F).

## Discussion

Up until now, chemotherapy has been a cornerstone of control measures against toxoplasmosis. The severe side effects of the currently available drug regimens create an urgent need to develop a new therapeutic agent with higher efficacy and fewer drawbacks.

Owing to the known antioxidant and anti-inflammatory effects of alginate, the current study tested it as an oral therapeutic agent for toxoplasmosis (Maciel et al. 2013; Rocha de Souza et al. 2007; Zhou et al. 2015). Oral route was chosen as alginate can preserve its properties in acidic media in the stomach (Draget and Taylor 2011). Besides, being a mucoadhesive polysaccharide increases its bioavailability and allows a reduction in the frequency of oral dosing (Bernkop-Schnürch et al. 2001). Alg-NP was prepared by the ionic gelation method, which is a single-step process that does not require any special equipment and results in a narrow particle size distribution (Paques et al. 2014). The characterization of Alg-NP prepared in this study showed a regular, smooth outline of the nanoparticles with a mean diameter of 70.53 ± 4.31 nm. This result goes with Gaafar et al. (2022), who used the same method in preparing the Alg-NP (Gaafar et al. 2022).

The results of the current study showed that treatment with Alg-NP significantly reduced the parasite burden and prolonged the survival time of experimentally infected mice with an improvement in histopathological derangements in the liver and spleen.

The survival time in mice treated with Alg-NP (subgroup IIb) reached 13 days with no significant difference compared to TMP-SMZ-treated mice (subgroup IIa). This could be explained by the significant reduction of the tachyzoite counts (83%R) and parasite viability (47%R) as detected in the peritoneal lavage fluid of mice treated with Alg-NP. These results were in accordance with Gaafar et al. (2014), who used chitosan nanoparticles in the treatment of toxoplasmosis (Gaafar et al. 2014).

Reduction in tissue-parasite load in the liver and spleen impression smears from Alg-NP-treated mice (79.12%R and 88.97%R, respectively) could explain the improvement of infection outcome and prolonged animal survival in subgroup IIb, even though statistical analysis was non-significant compared to TMP-SMZ-treated mice (subgroup IIa) (El-Temsahy et al. 2002). This non-significant difference places the newly tested formulation at nearly the same level of anti-toxoplasmosis activity as TMP-SMZ. However, Alg-NP showed a further step forward in this aspect due to its safety, as was noted in the present liver and kidney function assays.

Ultrastructural deformities in Alg-NP-treated tachyzoites could explain the decreased parasite counts owing to deranged parasite multiplication capability. Moreover, TEM demonstrated apoptotic activity in Alg-NP-treated tachyzoites, as was demonstrated in nuclear clefts and vacuolization of the cytoplasm. These results could be further explained based on the immunostimulant effect of the alginate formulation proven in the present study. It is worth mentioning that cytoplasmic vacuolization and haziness of cellular organelles were reported as signs of intracellular parasites’ response to treatment, a phenomenon known as autophagy, as was noted by Arafa et al. (2023).

Studies have documented the critical role of IFN-γ in resistance against intracellular parasites such as *Toxoplasma*. It limits the reproduction of the tachyzoites in the acute stage of the infection and restricts *Toxoplasma* development in the target cells, thus controlling infection (Suzuki et al. 2011; Takács et al. 2012).

In the current study, treatment of mice with Alg-NP (subgroup IIb) results in a significant rise in the level of IFN-γ reaching 763.8 ± 69.6 versus 538.3 ± 29 pg/ml in TMP-SMZ-treated mice (subgroup IIa). This might explain the protective potential of Alg-NP in toxoplasmosis and the reduction of the tachyzoite count in the peritoneal fluid. Studies showed that using different agents formulated with Alg-NP resulted in the stimulation of high levels of INF-γ in the sera of treated mice, which combined with antioxidative and immunomodulatory effects, caused prolonged survival in mice (Vetvicka and Fernandez-Botran 2018; Zhou et al. 2015). Furthermore, it was reported that mouse vaccination using Alg-NP as an adjuvant resulted in the induction of a high level of IFN-γ (Gaafar et al. 2022).

It is well known that *Toxoplasma* develops a strategy to bypass apoptosis to conserve the integrity of the host cells by blocking the apoptotic mitochondrial pathways of these cells by preventing over-induction of proinflammatory mediators produced by the innate immune system. This was executed by exploiting a signal transducer and activator of transcription 3 (STAT3), a protein that transmits signals for immune cell maturation, to downregulate IL-12 and TNF-α, which are pro-inflammatory mediators required for macrophage activation and nitric oxide production (Baron et al. 2010). The enhanced production of INF-γ by Alg-NP could go through the *Toxoplasma* apoptotic evading strategy. Most of the biological effects of INF-γ are mediated through STAT1, which in turn activates apoptosis (Matta et al. 2018).

The ability of Alg-NP to induce IFN-γ production could be explained by the presence of β-glucan, which is one of the most important components of alginate (Salmeán et al. 2017). It has been well established that β-glucan protects against infection with both bacteria and protozoa, as it can stimulate both humoral and cellular immunity (Vetvicka and Fernandez-Botran 2018). This immunostimulatory role of β-glucan is caused by receptor-mediated stimulation of various immune cells, including macrophages, leukocytes, and natural killer cells (Legentil et al. 2015). Furthermore, alginate is known to be rich in mannuronate residues, which can stimulate pro-inflammatory cytokine production and activate Toll-like receptors (Draget and Taylor 2011).

Examination of the H and E-stained liver and splenic sections of the infected, non-treated control subgroup Ic showed heavy infiltration by inflammatory cells with multiple necrotic foci. On the other hand, significant improvement in the histopathological changes in the Alg-NP-treated subgroup IIb was detected. Hepatic sections treated with Alg-NP showed preserved liver architecture with few necro-inflammatory foci. This could be explained by the reduced number of tachyzoites observed in the liver sections, as the tachyzoites are responsible for stimulating of an inflammatory immune response that causes damage and lysis of the host cell. Adding to the inflammatory response, tachyzoites can cause direct mechanical damage to the host cell, as mentioned by Legentil et al. (2015) and Unno et al. (2013) (Fuentes-Castro et al. 2017; Unno et al. 2013).

Splenic sections showed enhanced immune response by Alg-NP in the form of hyperplasia of lymphoid follicles with preserved white pulp and scanty tachyzoites that were surrounded by mild inflammatory cells. The lymphoid hyperplasia may indicate activation of the B-lymphocyte that controls the infection and causes a reduction in the parasite load (Fuentes-Castro et al. 2017). These results are in agreement with Hagras et al. (2022). It is worth noting that the high level of IFN-γ detected in the serum of Alg-NP-treated subgroup IIb can lead to the abovementioned histological amelioration.

In conclusion, the present work paved the way for Alg-NP as a promising, safe therapeutic agent against toxoplasmosis. It resulted in a significant prolongation of mouse survival and a significant reduction in parasite counts in the different tissues. Additionally, treated mice showed significant amelioration in the histopathological changes of both hepatic and splenic tissue, apparently caused by the high level of IFN-γ induced by the Alg-NP.

## Data Availability

No datasets were generated or analysed during the current study.
